# Features of Lymph Node Metastasis and Structural Recurrence in Papillary Thyroid Carcinoma Located in the Upper Portion of the Thyroid: A Retrospective Cohort Study

**DOI:** 10.3389/fendo.2021.793997

**Published:** 2022-01-25

**Authors:** Yu Heng, Siqi Feng, Zheyu Yang, Wei Cai, Weihua Qiu, Lei Tao

**Affiliations:** ^1^ ENT Institute and Department of Otorhinolaryngology, Eye & ENT Hospital, Fudan University, Shanghai, China; ^2^ Department of General Surgery, Liaoning Cancer Hospital & Institute, Shenyang, China; ^3^ Department of General Surgery, Ruijin Hospital, Shanghai Jiaotong University School of Medicine, Shanghai, China

**Keywords:** papillary thyroid carcinoma, lymph node metastasis, tumor location, treatment strategy, postoperative radioactive iodine

## Abstract

**Background:**

This study aims to reveal the features of lymph node metastasis (LNM) and recurrence in papillary thyroid carcinoma (PTC) tumors located in the upper portion of the thyroid.

**Methods:**

A total of 1075 PTC patients were retrospectively reviewed, including 314 patients with a tumor in the upper portion of the thyroid. Another 103 PTC patients with upper portion diagonsis from three clinical centers were included for external validation.

**Results:**

The results showed no difference between the patients with a tumor in the upper portion of the thyroid and those with a tumor in the non-upper portion in terms of overall LNM rates. However, patients with a tumor in the upper portion were significantly more prone to LLNM and exhibited a significantly worse recurrence outcome than those with a tumor in other subregions. Multivariate analysis showed that four factors—age no more than 40, maximum tumor diameter no less than1.0 cm, the presence of thyroid capsular invasion, and tumor with ipsilateral nodular goiter—were independent risk factors for LLNM of the tumor in the upper thyroid. A predictive risk-scoring model was established based on these factors.

**Conclusions:**

Patients with PTC located in the upper portion may have an exclusive lymphatic drainage pathway to the lateral neck region and are more prone to suffer from LLNM and tumor recurrence than those with a tumor located in other subregions. A new postoperative strategy selection flow chart was established based on our newly created risk-scoring model that can effectively predict the individualized possibility of LLNM for PTC patients with a tumor in the upper portion.

## Introduction

The incidence of thyroid cancer is on the rise, and the disease is projected to become the fourth leading type of cancer worldwide (1). Although over screening and increased diagnosis might play a role in certain parts of the world, the research has indicated that other areas could be experiencing a true increase in incidence due to elevated exposure risks (2, 3). Papillary thyroid cancer (PTC), the most common pathological type of thyroid cancer, has a better prognosis, and researchers have proposed the need for active surveillance of suspected thyroid cancer instead of aggressive surgical intervention, which has been disputed (4, 5). Although the mortality rate associated with PTC has not changed (disease-specific mortality at 10 years is less than 5%), patients with local advanced thyroid cancer still face a high risk of recurrence and distant metastasis (6–8). The main difficulty of local advanced thyroid cancer is the diagnosis and treatment of lymph node metastasis (LNM), particularly LNM in the lateral neck (N1b).

According to the literature, PTC involves cervical lymph node metastasis in 20%–50% of patients with macrometastasis and in up to 90% of patients with micrometastasis detected using sensitive detection methods (9–12). LNM of PTC occurs in a stepwise fashion. Spreading from the thyroid gland, the central and lateral lymph node compartments on the ipsilateral side of the thyroid tumor represent the first echelons of lymphatic drainage, followed by the mediastinal and contralateral lateral lymph node compartments (13, 14). In a previous study, our team found that the region of PTC LNM was significantly associated with the location of the primary tumor (15). N1b metastasis, which thyroid surgeons focused on, was highly correlated with the occurrence of tumors in the upper portion of the thyroid. According to the 2015 American Thyroid Association (ATA)-modified initial risk stratification system (RSS), LNM (N1) together with extrathyroidal extension (ETE), vascular invasion, BRAF mutation, etc. were the core factors leading to postoperative recurrence in PTC patients. Although the ATA-RSS was used to comprehensively assess risk factors for recurrence in PTC patients, the impact of the primary tumor site on prognosis was missing.

Several researchers have focused on the association between tumor location and the region of LNM (16, 17), indicating that the upper portion of the thyroid could be a vital risk factor for lateral LNM (LLNM) in PTC patients. However, what caused us to think was, when we only focused on the upper portion PTC, which risky group of these patients would suffer LLNM and would they have a higher risk of recurrence. In the present study, we verified the association between the primary tumor location and LNM regions in PTC and explored the risk factors for N1b metastasis of tumors in the upper portion of the thyroid. Therefore, in addition to promoting individualized treatment in clinical practice, we provide an important research basis for further clarifying the LNM patterns in thyroid cancer.

## Methods

### Patient Cohort

The patient cohort comprised 1179 newly diagnosed primary PTC patients who underwent thyroidectomy at the Department of General Surgery, Ruijin Hospital, Shanghai Jiao Tong University School of Medicine and the Department of Otorhinolaryngology, Head and Neck Surgery at the Eye, Ear, Nose, and Throat (EENT) Hospital of Fudan University in the period June 2017–June 2019. Patients with poorly differentiated thyroid cancer pathological diagnosis, initial distant metastasis, irregular follow-up visits, having no lymph nodes removed or incomplete laboratory and pathological results were excluded. After exclusion, 1,075 patients who had pathological PTC and who underwent thyroidectomy and lymph node dissection were studied, including 812 patients from Ruijin Hospital and 263 patients from EENT Hospital. In addition, we included 103 PTC patients from three clinical centers(Ruijin Hospital 46, Liaoning Cancer Hospital&Institute 35, EENT Hospital 22) with upper portion diagnosis in the period Jan 2020-Dec 2020 for external validition. The patient cohort used in this study is consistent with that in a study on skip metastasis by our team that explored different clinical problems.

### Surgical Methods and Pathological Approach

Preoperative data sources including basic clinical information, ultrasound examination(US) and fifine-needle aspiration (FNA) were collected from Electronic Medical Records System for further analysis. Preoperative US and US-guide FNA were performed strictly according to Thyroid Imaging Reporting and Data System(TI-RADS). Tumor location was categorized as upper portion, middle portion, lower portion, isthmus and diffuse PTC based on the preoperative ultrasound report from experienced ultrasound doctors and the findings obtained during the operation or pathological description. The thyroid glands were bisected into three equal volumes (upper portion, middle portion and lower portion) according to the consensus of most clinical medical center. Tumors with a maximum diameter of more than 2cm that were primarily located in the upper portion and did not exceed the lower 1/3 thyroid gland were also defined as upper portion tumor in this study. The same criteria were used for intraoperative description and postoperative pathology in classification of tumor location. In addition to thyroid and parathyroid glands, description of central and lateral lymph nodes were also included. All patients enrolled were identified as T_0-4_N_0-1b_M_0_ according to the 2015 American Joint Committee on Cancer (AJCC) Tumor Node Metastasis (TNM) staging system. Surgical procedures include total thyroidectomy and thyroid lobectomy with routine central compartment lymph node dissection (LND), and lateral LND including ipsilateral levels IIa, III, IV, VI in patients with LLNM. LLNM in this study included skip metastasis. Patients with unilateral lesions without LLNM underwent lobotomy, patients with bilateral lesions without LLNM underwent total thyroidectomy, both of which performed preventive/therapeutic CLND routinely. All patients with LLNM underwent modified radical thyroidectomy, including total thyroidectomy and lymph node dissection of central and lateral regions.

All acquired specimens were examined by two or more board-certified pathologists from Shanghai Ruijin Hospital, Shanghai EENT Hospital, Liaoning Cancer Hospital&Institute. Pathological features analyzed were pathological type of tumor, type of the surrounding thyroid tissues, tumor size, multifocality (more than one lesion in unilateral thyroid lobe) and lymph node metastasis.

### Criteria for Recurrence and Follow-Up

In this study, recurrence was defined as structural recurrence, excluding new lesions in residual thyroid gland, included recurrence in thyroidectomy bed, lymph nodes, and distant site. None of the patients enrolled in the study had distant metastasis during follow-up period. Recurrence was defined as structural recurrence after completion of initial treatment, identified using imaging modalities, i.e. US examination and/or radioactive iodine-131(RAI) whole-body scan imaging, followed by cytological or histological confirmation, regardless of serum levels of Tg.

All enrolled patients underwent short-term postoperative follow-up at the 1st, 3rd and 6th months after surgery, including thyroid function, parathyroid function, electrolytes and other hematological indicators, as well as physical examinations such as surgical incision, voice and drinking cough tests. Then follow-up was performed at 6-month intervals, including thyroid function, parathyroid function, and ultrasound. Patients regularly followed up for more than 1 year were included in the study subjects for retrospective analysis.

### Statistical Analysis

Chi-square test and independent t-test were conducted for categorical variables and continuous variables respectively. Univariate and multivariate analyses were conducted for screening risk variables that were significantly associated with lateral lymph node metastases. Kaplan-Meier method and log-rank test were used to compare recurrence-free survival estimates. P-value <0.05 was considered to indicate a statistically significant difference, and statistical analyses were conducted using the SPSS 24.0 package (SPSS Inc., Chicago, IL, USA). Variables of which the p-value < 0.05 from the univariate logistic regression were then used for multivariate logistic regression to construct a risk prediction model – Nomogram, in R software (ver. 3.5.1, R Development Core Team). The discrimination and consensus degree of our newly-established predictive model were tested through the receiver operating characteristic (ROC) curve, the calibration curve, and the concordance index (C-index).

### Ethical Statement

This study was approved by the Institutional Ethics Committee of the Eye and ENT Hospital of Fudan University and Ruijin Hospital, Shanghai Jiao Tong University School of Medicine, and was also approved by Chinese Clinical Trial (ChiCTR2100043353). All participants gave informed consent to take part in the study after full explanation of the purpose and nature of all procedures used.

## Results

### Demographics, Clinicopathological Characteristics of Patients in the Cohort

A total of 1,075 PTC patients including 381 males (35.4%) and 694 females (64.6%) diagnosed with PTC in our institution. Mean age was 42.9 years with a range of 18-71 years. Through preoperative US detection and postoperative pathological results, 314(29.2%) had tumor located in the upper portion, while 322(30.0), 357(33.2), 57(5.3), 25(2.3) in middle, lower, isthmus portion and diffuse PTC, respectively. In addition, 236 (21.9%) were confirmed to have bilateral PTC, and 580 (53.9%) were ultimately confirmed to have LNM by postoperative pathology. In patients with LNM, 37 patients(6.4%) were diagnosed with skip metastasis, while 383(66.0%) with CLNM and 197(34.0%) with LLNM. The mean harvested central lymph nodes in skip, CLNM, LLNM were 7.9, 6.7, 8.1, respectively, and the mean number of positive central lymph nodes in CLNM, LLNM were 3.3, 4.1, respectively (**Table 1**). We also analyzed the characteristics of tumor location and LNM. The result showed that there was no difference between upper portion group and non-upper portion group in terms of cervical lymph node metastasis including both CLNM and LLNM (56.7% vs 52.8%, p-value =0.250). However, patients with upper portion tumors were significantly more prone to LLNM than those with tumor located in other subregions (25.8% and 15.2%, respectively, p-value <0.01. Shown in **Table 2**).

**Table 1:** Demographics and clinical Characteristics of the cohort.

Variable	Value(%)
**Age**	
** mean**	42.9
**Gender**	
** male**	381 (35.4)
** female**	694 (64.6)
**Size of largest lesion (US)**	
** mean**	0.93
** medium**	0.9
**Tumor Location**	
** Upper portion**	314 (29.2)
** Middle portion**	322 (30.0)
** Lower portion**	357 (33.2)
** Isthmus**	57 (5.3)
** Diffuse PTC**	25 (2.3)
**Bilateral disease**	236 (21.9)
**Negative LNM**	495 (46.1)
** Mean harvested central lymph nodes**	5.1
**LNM**	580 (53.9)
** Mean harvested central lymph nodes**	7.9
** CLNM**	383 (66.0)
** Mean harvested central lymph nodes**	6.7
** Mean positive central lymph nodes**	3.3
**LLNM**	197 (34.0)
** Mean harvested central lymph nodes**	8.1
** Mean positive central lymph nodes**	4.1
**Recurrence**	46 in 1075 (4.3)
** No LLNM**	24 in 761 (3.2)
** LLNM recurrence**	15 (62.5)
** CLNM recurrence**	6 (25.0)
** Recurrence in situ**	3 (12.5)
**LLNM**	22 in 314 (7.0)
** LLNM recurrence**	14 (63.6)
** CLNM recurrence**	6 (27.3)
** Recurrence in situ**	2 (9.1)

PTC, papillary thyroid carcinoma; LNM, lymph node metastasis; CLNM, central lymph node metastasis; LLNM, lateral lymph node metastasis.

**Table 2:** Characteristics of tumor location and LNM in PTC patients.

	UP LOC	%	non-UP LOC	%	p-value
**LNM group**	178	56.7	402	52.8	
**non-LNM group**	136	43.3	359	47.2	0.250
**CLNM group**	97	30.9	286	37.6	
**non-CLNM group**	217	69.1	475	62.4	0.037
**LLNM group**	81	25.8	116	15.2	
**non-LLNM group**	233	74.2	645	84.8	<0.01

PTC, papillary thyroid carcinoma; UP LOC, tumors located in the upper portion of the thyroid; non-UP LOC, tumors located in the non-upper portion of the thyroid; LNM, lymph node metastasis; CLNM, central lymph nodemetastasis; LLNM, lateral lymph node metastasis.

### Clinicopathological Characteristics and LLNM of Patients With Upper Portion Tumors

**Figure 1:**
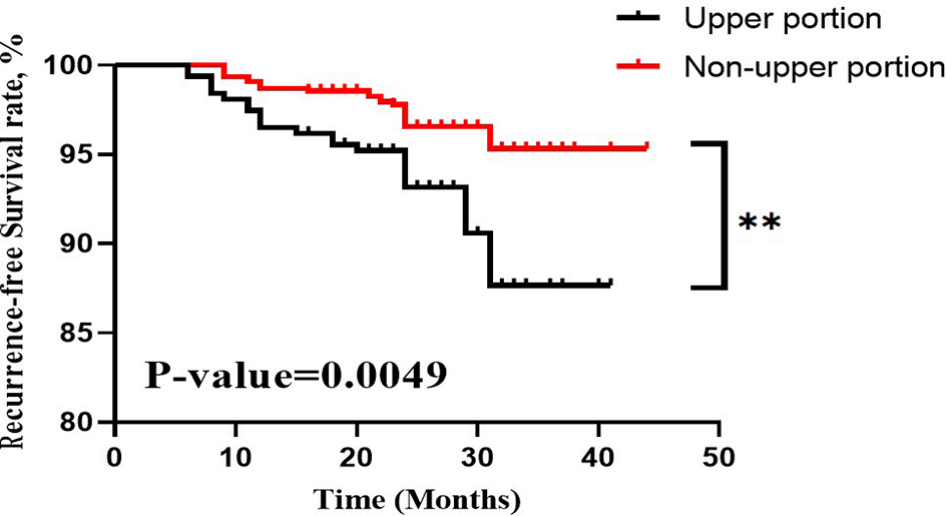
RFS curves of PTC patients stratified by tumor located in upper portion or not. RFS, recurrence-free survival; PTC, papillary thyroid carcinoma. **, p-value <0.01.

Among the 314 PTC patients with upper portion tumors, 101 (32.2%) were male and 213 (67.8%) were female. The mean age was 42.70 ± 12.55 years (ranging from 19 to 75). 81 (25.8%) patients were considered as having LLNM in our research. Main clinicopathological characteristics of patients with PTC located in upper portion were shown in **Table 3**. The tumor sizes for patients with positive LLNM were significantly bigger than those in Non-LLNM Group ((1.33 ± 0.73) and (0.77 ± 0.51), respectively, p-value =0.000). In addition, age no more than 40 years old, female, the presence of thyroid capsular invasion (TCI) and tumor with ipsilateral nodular goiter (NG) were more commonly seen in positive LLNM Group (P-value =0.000, 0.014, 0.000 and 0.005, respectively). A Kaplan-Meier analysis and the log-rank test were also carried out to evaluate the difference in postoperative recurrence-free survival (RFS) between patient with tumor located in upper portion or not. The result showed that patients with upper portion tumor exhibited significantly worse recurrence outcome than those with tumor located in other subregions of thyroid (p-value =0.0049, **Figure 1**).

**Table 3:** The clinicopathological characteristics of patients with tumor located in upper portion.

	All Patients		Non-LLNM Group		LLNM Group		P value
	n=314	%	n=233	%	n=81	%	
**BMI(mean ± SD)**	23.66 ± 3.67		23.68 ± 3.64		23.61 ± 3.75		0.873
**Maximum tumor diameter(mean ± SD)**	0.91 ± 0.62		0.77 ± 0.51		1.33 ± 0.73		0.000
**Age**							0.000
>40	157	50.0	130	55.8	27	33.3	
<=40	157	50.0	103	44.2	54	66.7	
**Gender**							0.014
Male	101	32.2	66	28.3	35	43.2	
Female	213	67.8	167	71.7	46	56.8	
**Thyroid capsular invasion**							0.000
No	170	54.1	151	64.8	19	23.5	
Yes	144	45.9	82	35.2	62	76.5	
**Bilateral disease**							0.079
Absent	236	75.2	181	77.7	55	67.9	
Present	78	24.8	52	22.3	26	32.1	
**Multifocality**							0.059
Absent	209	66.6	162	69.5	47	58.0	
Present	105	33.4	71	30.5	34	42.0	
**PTC with ipsilateral Hashimoto thyroiditis**							0.083
No	246	78.3	177	76.0	69	85.2	
Yes	68	21.7	56	24.0	12	14.8	
**PTC with ipsilateral nodular goiter**							0.005
No	221	70.4	174	74.7	47	58.0	
Yes	93	29.6	59	25.3	34	42.0	

### Construction of the Risk-Scoring Model for Predicting LLNM in PTC Patients With Tumor Located in Upper Portion

**Figure 2:**
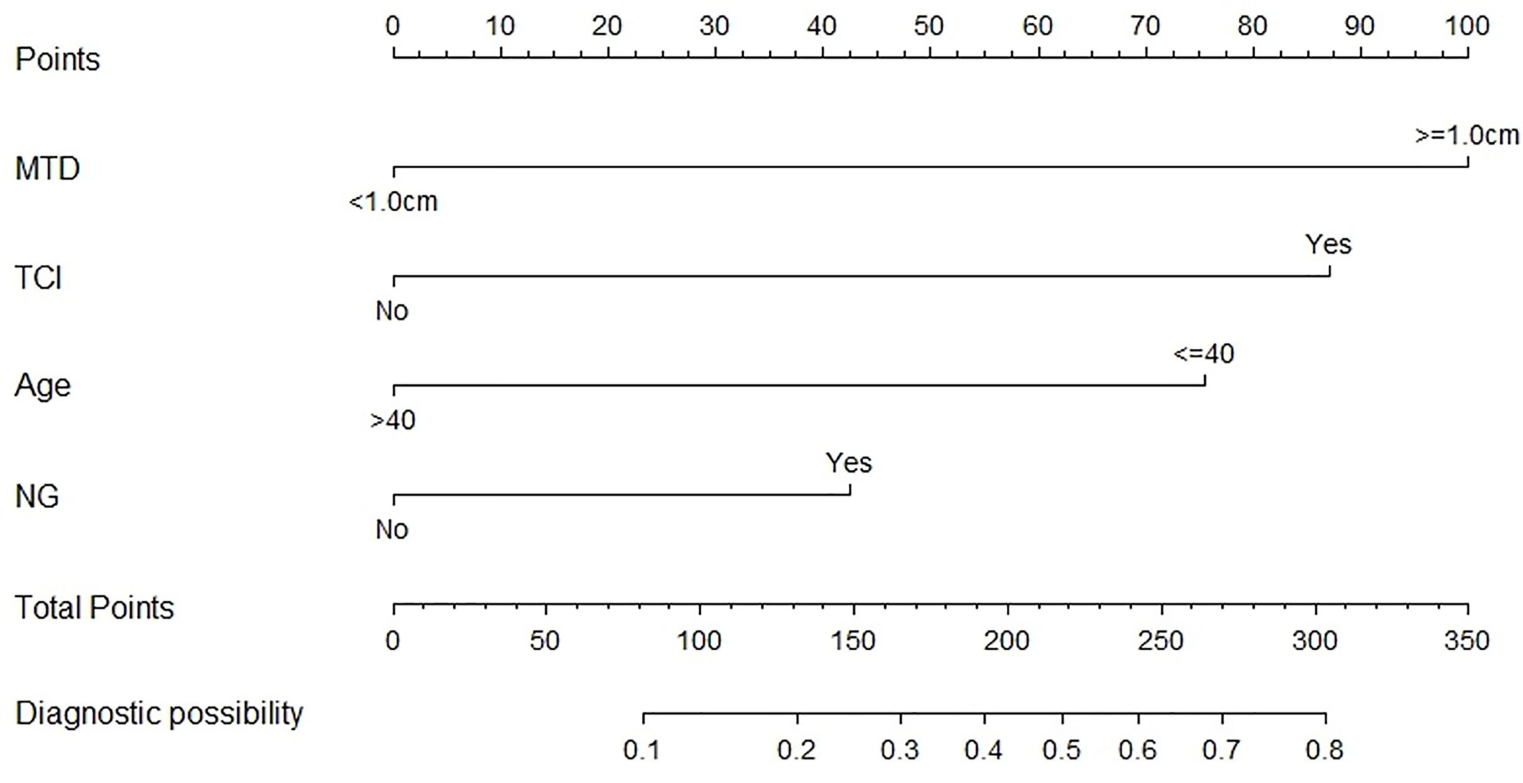
The risk-scoring model for predicting LLNM risk in PTC patients with tumor located in upper portion.

Both demographic and clinicopathological characteristics were analyzed by the logistic univariate analysis to evaluate their correlation with LLNM in PTC patients with tumor located in upper portion. Factors with P-values <0.05 were incorporated into multivariate regression analysis for further screening. Finally, four factors including age no more than 40 years old, maximum tumor diameter no less than1.0cm, the presence of TCI and NG, were recognized as independent risk factors of LLNM in these patients (shown in **Table 4**). Then a nomogram-based risk-scoring model incorporating the above-mentioned four factors was established to quantitatively assess the risk of LLNM in patients with upper portion tumors (shown in **Figure 2**).

**Table 4:** Univariate and multivariate analyses for tumor located in upper portion.

	Univariate analysis		Multivariate analysis	
	Hazard ratio (95% CI)	P value	Hazard ratio (95% CI)	P value
**Factors selected**				
**Age**		***0.001***		***0.000***
>40 vs. <=40	0.396 (0.233-0.673)		0.314 (0.167-0.589)	
**Sex**		***0.014***		0.138
Male vs. Female	1.925 (1.140-3.251)		1.658 (0.850-3.235)	
**Thyroid capsular invasion**		***0.000***		***0.000***
Yes vs. No	6.009 (3.364-10.733)		4.127 (2.154-7.904)	
**Bilateral disease**		0.081		
Present vs. Absent	1.645 (0.941-2.878)			
**Maximum tumor diameter (MTD)**		***0.000***		***0.000***
>= 1.0cm vs. < 1.0cm	6.099 (3.515-10.584)		4.654 (2.498-8.674)	
**PTC with ipsilateral Hashimoto thyroiditis**		0.086		
Yes vs. No	0.550 (0.278-1.088)			
**PTC with ipsilateral nodular goiter**		***0.005***		***0.017***
Yes vs. No	2.133 (1.255-3.628)		2.197 (1.150-4.195)	
**Multifocality**		0.060		
Present vs. Absent	1.651 (0.979-2.782)			

Bold value, p-value <0.05.

### Evaluation and Validation of the Risk-Scoring Model

**Figure 3:**
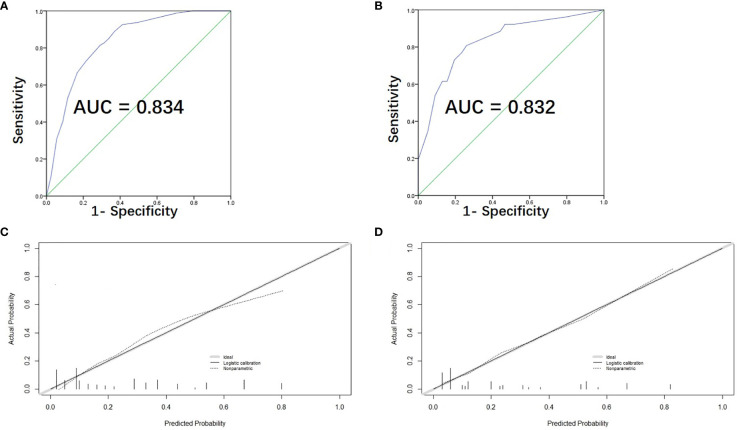
Assessment, and validation of the predictive model. **(A)** The ROC curve and AUC of the risk-scoring model; ROC receiver operating characteristics. **(B)** The ROC curve and AUC for external validation group. **(C)** The calibration curve of the risk-scoring model for predicting LLNM risk. Actual probability is plotted on the y-axis, and nomogram predicted probability on the x-axis. **(D)** The calibration curve for external validation group.

We used the C-index and the calibration plot to assess the precision of our newly created scoring model. The ROC curve was exhibited (**Figure 3A**) and the C-index of our model was found to be 0.834 (95% CI, 0.787-0.881). The calibration plot also showed the actual and estimated probability of LNM were in fair agreement (**Figure 3C**), both indicating satisfactory discrimination and accuracy of our model’s prediction ability.

Furthermore, 103 PTC patients with tumor located in upper portion from the two medical centers were used as external validation group for assessing the predictive accuracy of our model. The ROC curve of the validation group was shown in **Figure 3B** and the C-index turned out to be 0.832 (95% CI, 0.738-0.926) for external validation group. The calibration curve of the validation group was also displayed in **Figure 3D**, showing excellent accordance between the predicted and the actual presence of LLNM.

### Novel Risk Stratification of LLNM for PTC Patients With Tumor Located in Upper Portion

Each individual can get a total LLNM risk point by summing up the risk points of each factors based on our risk-scoring model. In according to the distribution characteristic of the total risk points, the cutoff value was chosen to stratify upper portion PTC patients into three subgroups:

1. Patients with total LLNM risk point of <=50 were defined as low-risk group,
2. Patients with total LLNM risk point of 50 - 100 were classified as moderate-risk group.
3. Patients with total LLNM risk point of >=100 were classified as high-risk group.

Chi-square test showed a significant difference among the three subgroups in terms of lateral lymph node involvement (3.8%,22.7% and 51.5% for low-, moderate- and high-risk group, respectively, P-value =0.000, shown in **Table 5**).

**Table 5:** Risk stratification of PTC patients located in upper portion.

	Training Group (N=314)			P value	Validation Group (N=103)			P value
	Low risk	Moderate risk	High risk		Low risk	Moderate risk	High risk	
	(N = 52, %)	(N = 194, %)	(N = 68, %)		(N = 26, %)	(N = 16, %)	(N = 61, %)	
**Negative LLNM**	50 (96.2)	150 (77.3)	33 (48.5)	0.000	25 (96.2)	13 (81.3)	39 (63.9)	0.005
**Positive LLNM**	2 **(3.8)**	44 **(22.7)**	35 **(51.5)**		1 **(3.8)**	3 **(18.8)**	22 **(36.1)**	

Bold value, p-value <0.05.

The result was also confirmed by patients in the external validation group (3.8% (1 in 26), 18.8% (3 in 16) and 36.1% (22 in 61) for high-risk, moderate-risk and low-risk, respectively, P-value = 0.005, shown in **Table 5**).

## Discussion

Many predicting models have been proposed to assess the risk of recurrence in PTC patients, while ATA-RSS is the most widely accepted and clinically used system (18–20). ATA constantly refined its risk stratification models based on the latest research, as the ATA 2015-RSS were revised from its previous version in aspects including lymph nodes metastasis and BRAF mutation, etc. In clinical practice, our team found that previous studies of risk stratification models have ignored the effect of primary tumor location on recurrence in PTC patients. However, in our previous study, we found that tumor location was closely related to different lymph node metastasis patterns, indicating that the difference in primary tumor sites might have effect on the prognosis of PTC patients, especially the recurrence risk, hence further research on tumor location were needed. In the present study, we analyzed the data of 1075 PTC patients and made comparisons on characteristics of LLNM and recurrence between tumors with different primary locations. The results showed that tumor located in upper portion was more prone to LLNM and had significantly higher risk of recurrence in PTC patients, which aroused our interest in further exploring the characteristics of lymph node metastases in upper portion tumors.

Besides the common stepwise metastatic fashion: firstly metastasizes to the central compartment and then to the ipsilateral lateral region, some researches have revealed the particular lymphatic drainage pathway of upper portion tumor by anatomical research (21). Dou et al. (22) reported that tumor located in the upper portion may have an exclusive drainage pathway to the lateral lymph node regions and lateral neck dissection should be evaluated more meticulously for these patients. Here in our research, among the 81 patients with LLNM, 19 (23.5%) were skip metastasis, which substantiates the relative high risk of lateral neck region involvement in patients with primary upper portion tumor. In view of the exclusive lymphatic drainage and the high probability of LLNM in patients with upper portion tumor, the management of lateral neck region need to be critically evaluated during the operation. For those who had LLNM, if the extent of initial tumor resection does not include lateral neck region, a second operation is almost inevitable. However, generalization of prophylactic lateral lymph node dissection for patients with upper portion tumor is also not recommended by most clinical centers in view of the relatively high occurrence of postoperative complications including chyle leakage, postoperative bleeding, nerve injury, shoulder ache, and limited mobility (23). Thus, it is very important to meticulously assess whether lateral lymph node involvement exist or not. Literature that quantitatively assesses the LLNM risk among PTC patients with upper portion tumor is vacant.

So here in our research, we focus on PTC of upper portion origin and sought to explore the related risk factors of LLNM in patients with PTC located in upper portion. As a result, a risk-scoring model were created based on four selected factors: age no more than 40 years old, maximum tumor diameter no less than 1.0cm, the presence of thyroid capsular invasion (TCI) and tumor with ipsilateral nodular goiter (NG), to quantitatively measure the LLNM risk in PTC patients with upper portion tumor.

Larger tumor volume has been reported to be associated with both central and lateral lymph node involvement in PTC patients (24–27). Our research focused on patients with upper portion tumor and found that LLNM are more commonly occurred in those with maximum tumor diameter no less than 1.0cm, which is consistent with existing studies on all PTC patients. However, the association between patient’s age and LLNM risk is rarely reported. Our study demonstrated that young patients are prone to LLNM among all patients with upper portion tumor. Interestingly, the risk factor “tumor with ipsilateral nodular goiter (NG)” was not commonly used in most existing literatures. One of our previous study tumor has proven that tumor with ipsilateral nodular goiter is positively related with LLNM, and this result has been confirmed again in patients with PTC located in the upper portion in our current research, implying that the coexistence of nodular goiter portends a more aggressive tumor (22).

**Figure 4:**
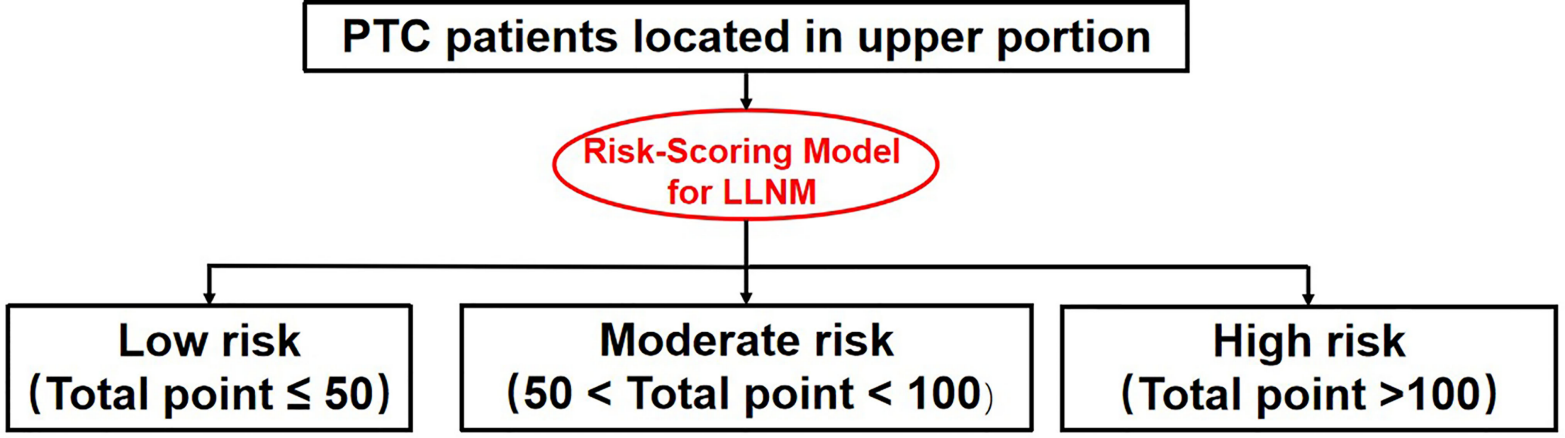
Risk stratification flow chart for PTC patients with tumor located in upper portion.

Although not every last upper portion tumor had the likelihood of LNM, this part of tumors did tend to accompany with LLNM because of the lymphatic drainage pathways. Upper portion tumors were more prone to suffer from LLNM, in turns meaning a higher risk of recurrence (6, 18–20), which logical relationship was consistent with the clinical manifestations we observed. Therefore, it is of great clinical value to screen out the high-risk group of LLNM in upper portion tumors. The upper portion tumor was considered as an independent risk factor, and then a secondary risk stratification was conducted for this part of PTC patients, so as to more accurately stratify the tumor risk and develop individual treatment strategies. Patients with upper portion tumor were divided into three subgroup with significantly different level of LLNM risk by our newly-created risk-scoring model. In view of the extremely low incidence rate of positive LLNM, close follow-up is sufficient for patients that categorized into low risk subgroup, and other postoperative interventions are unnecessary; When making decisions for those with moderate risk, patient’s preference and clinician’s judgment should be comprehensively considered, and close follow-up or adjuvant radioactive iodine are all available options. However, for patients in the high-risk subgroup in which the incidence of LLNM reaches up to 51.5%, prophylactic adjuvant radioactive iodine is recommended. A detailed risk stratification flow chart is shown in **Figure 4**, which help us stratify the LLNM risk in PTC patients with upper portion tumor and develop individual treatment strategies more accurately.

## Conclusions

Patients with PTC located in upper portion may have an exclusive lymphatic drainage pathway to the lateral neck region and are more prone to suffer from LLNM and tumor recurrence than those with tumor located in other subregions. A new postoperative strategy selection flow chart for predicting LLNM was also established.

## Limitations

There are several potential limitations in this study. First, the sample size of our study is not large enough. Second, for patients enrolled in our study were all diagnosed as PTC after June 2017, the follow-up time was not long enough. Third, the retrospective nature of our study means that nonrandomized features are inevitably produced. Thus, more reliable multicentric, large sample, prospective, randomized controlled studies are expected to validate our conclusions in the future.

## Data Availability Statement

The raw data supporting the conclusions of this article will be made available by the authors, without undue reservation.

## Ethics Statement

This study was approved by the Institutional Ethics Committee of the Eye and ENT Hospital of Fudan University and Ruijin Hospital, Shanghai Jiao Tong University School of Medicine. The patients/participants provided their written informed consent to participate in this study.

## Author Contributions

The article was written by YH, SF, and ZY and they contributed equally to this work. LT, WQ, and WC provided guidance to the manuscript preparation. All authors have approved the final version of the editorial.

## Funding

This research was supported by the Science and Technology Innovation Project of Shanghai Shenkang Hospital Clinical Development Center under Grant [SHDC2020CR6011, SHDC12015114]; the Science and Technology Commission of Shanghai Municipality under Grant [16411950100]; the National Natural Science Foundation of China under Grant [81772878, 30801283, 30972691]; the Shanghai Science and Technology Development Funds under Grant [20Y11902200, 09QA1401000, 10QA1405900]; the Training Program of the Excellent Young Talents of Shanghai Municipal Health System under Grant [XYQ2011055, XYQ2011015]; and the Shanghai Municipal Science and Technology Foundation under Grant [11JC1410802].

## Conflict of Interest

The authors declare that the research was conducted in the absence of any commercial or financial relationships that could be construed as a potential conflict of interest.

## Publisher’s Note

All claims expressed in this article are solely those of the authors and do not necessarily represent those of their affiliated organizations, or those of the publisher, the editors and the reviewers. Any product that may be evaluated in this article, or claim that may be made by its manufacturer, is not guaranteed or endorsed by the publisher.
